# Predictors of intention to leave and actual turnover among midwives — A three-year nationwide longitudinal study in Swedish healthcare

**DOI:** 10.1016/j.eurox.2026.100458

**Published:** 2026-03-27

**Authors:** Marcus Praetorius Björk, Rikke Damkjær Maimburg, Lena Henriksen, Malin Hansson

**Affiliations:** a Region Västra Götaland, Research, Education, Development and Innovation, Primary Health Care, Gothenburg, Sweden; b School of Public Health and Community Medicine, Institute of Medicine, Sahlgrenska Academy, University of Gothenburg, Gothenburg, Sweden; c Department of occupational Medicine, Aarhus University Hospital, Aarhus, Denmark; d Department of Clinical Medicine, Aarhus University, Aarhus, Denmark; e University College of Northern Denmark, School of Midwifery, Aalborg, Denmark; f School of Nursing and Midwifery, Western Sydney University, Sydney, Australia; g Department of Nursing and Health Promotion, Oslo Metropolitan University, P.O. Box 4 St. Olavs plass, Oslo 0130, Norway; h Institute of Health and Care Sciences, Sahlgrenska Academy, Gothenburg University, Gothenburg, Sweden

**Keywords:** Intention to leave, Midwifery, Salary satisfaction, Turnover

## Abstract

**Objectives:**

This study explored midwives’ intentions to leave their position or the profession, actual turnover, and salary satisfaction over a three-year period. It also examined differences between primary and hospital care and identified predictors of turnover.

**Methods:**

A longitudinal survey was conducted with 478 midwives in 2020 and 2023. Measures included intention to leave, salary satisfaction, and actual turnover. Predictors were analysed using linear and logistic regression models.

**Results:**

By 2023, 39% of midwives expressed an intention to leave the profession. Intention to leave their current position decreased slightly over time, while salary satisfaction increased. Significant predictors of intention to leave included baseline intention (β = −0.698, p < .001), salary satisfaction (β = −0.128, p < .001), and professional experience (β = −0.113, p < .001). Turnover was higher in primary care (OR = 2.83, p < .001). Salary satisfaction was also linked to actual turnover. Care setting did not predict intention to leave.

**Conclusion:**

Efforts to improve working conditions and ensure fair compensation are key to retaining midwives and maintaining quality care for mothers and newborns.

## 1 Introduction

Midwives have a crucial role in health care that frequently demands navigating professional expectations, acute situations, emotional labour, and physical strain, often in resource-constrained settings [1], [2], [3]. Former studies have shown these challenges may imply midwives feeling unable to deliver the care they aim to provide [4], [5]. Moreover, workplace conditions significantly impact job satisfaction and retention. Factors such as adequate staffing, managerial support, and opportunities for professional development are tied to workforce stability [5], [6], [7]. Midwives working in supportive environments report higher levels of job satisfaction and are less likely to leave the profession [8], [9]. Research shows that a lack of structured mentorship, limited organizational support, and the stressful transition from education to practice are major barriers to retention during the initial years of practice [10], [11], [12], [13]. Interestingly, previous research reveals both commonalities and variations across countries regarding midwives’ intentions to leave, turnover, and job satisfaction. While recurring themes such as workload, work-life balance, and organisational support are evident across contexts, differences emerge in the specific factors influencing these outcomes, reflecting variations in cultural contexts, healthcare systems, professional roles and autonomy [2], [14], [15], [16].

In recent years, a growing body of research has highlighted the challenges midwives face in their perceived work environment [4], [6], [17], [18]. Further research in adjacent professional groups, such as nurses with specialised critical-care training, shows that individuals with greater autonomy and access to organisational resources may experience stronger empowerment and higher job satisfaction [19], [20]. This comparison highlights the importance of workplace context and professional support when examining predictors of retention also among midwives. A literature review on midwives indicates that working hours, workload, and work schedules are factors that influence midwives' job satisfaction [21]. However, limited research has examined midwives’ intention to leave and turnover in longitudinal designs and in relation to salary and level of care. Importantly, turnover intentions represent conceptually distinct constructs. Research in organisational psychology and nursing workforce studies consistently distinguishes between intention to leave the current position and intention to leave the profession, as they involve different psychological processes, forms of commitment, and predictors [22], [23], [24]. In midwifery, these constructs also show different determinants [10]. Distinguishing between them is therefore essential when interpreting workforce instability.

Salary satisfaction is a possible mediator of job satisfaction and can impact employee’s intentions to leave [25], [26]. The relationship between salary satisfaction and intentions to leave can be theoretically interpreted through Psychological Contract Theory and Social Exchange Theory. Psychological Contract Theory argues that compensation is a core element of employees’ perceived obligations from the employer, and unmet salary expectations are experienced as a breach of this contract, which reliably predicts reduced commitment and increased turnover intentions [27], [28]. Complementing this, Social Exchange Theory conceptualises employment as a reciprocal relationship; when employees perceive inadequate financial return, they reduce their reciprocation, increasing the likelihood of withdrawal and intention to leave [29], [30]. Together, these frameworks provide a theoretical foundation for understanding why dissatisfaction with salary may erode organisational attachment and heighten intentions to leave. Empirical research supports these theoretical perspectives, indicating that perceived fairness, salary adequacy and fulfilment of financial expectations influence retention independent of other workplace challenges [31], [32].

In Sweden, midwives constitute a specialised and highly educated professional group with responsibilities spanning reproductive, maternal and sexual health. Of approximately 8000 individuals holding a midwifery degree, around one quarter are not currently working as midwives within the healthcare sector, reflecting challenges in retention and career sustainability [33]. Research on turnover intentions among midwives has primarily focused on hospital-based practice, whereas considerably less is known about midwives working in primary care [15], [34].

Primary care midwives play a central role in the Swedish system, providing youth clinic and antenatal care, postpartum follow-up, contraceptive counselling and long-term continuity-based sexual and reproductive healthcare thoughout the life course. Their work is typically scheduled, relational, and sustained over longer periods. In contrast, hospital-based midwives focus on intrapartum care, supporting physiological birth and acute obstetric events and immediate postpartum support. This work environment is often fast-paced and unpredictable, requiring rapid clinical decision-making and close interdisciplinary collaboration with obstetricians and other healthcare professionals.

These structural differences create distinct professional realities. While primary care midwives often work within structured and continuity-based care pathways, hospital-based midwives navigate high-intensity environments characterised by unpredictable workloads, acute obstetric events and frequent interdisciplinary coordination. These contrasting conditions may shape job satisfaction and turnover intentions in different ways. Despite these important contrasts, few studies have examined differences in turnover intentions or actual turnover between primary and hospital care settings. Understanding these contextual characteristics is therefore essential to interpreting retention patterns and identifying targeted strategies to support the midwifery workforce in both sectors.

The present analysis forms part of the nationwide GoodWEM project (Good Work Environment for Midwives) [5], [6], [8], [35], [36], [37], a longitudinal initiative examining organisational and psychosocial conditions shaping midwives’ professional roles, wellbeing and retention in Sweden. The baseline survey was conducted in 2020 among 5076 invited midwives (41% response rate, n = 2060), followed by a 2023 survey distributed to 4398 midwives (44% response rate, n = 1916).

Despite increasing attention to midwives’ retention internationally, little is known about how salary satisfaction interacts with organisational conditions to predict either turnover intentions or actual turnover. Evidence is particularly limited regarding how these predictors differ between primary and hospital care settings, which represent distinct professional and organisational environments.

Therefore, the aim of this study was to analyse midwives' considerations regarding leaving their position, considerations of leaving the profession, actual turnover, and salary satisfaction over three years in Sweden. A secondary aim was to explore differences between primary and hospital care and identify predictors of turnover.

## 2 Methods

### 2.1 Study design and participants

This longitudinal cohort study analysed data from midwives who participated in both the 2020 and 2023 waves of the GoodWEM project. A total of 478 midwives were included after excluding participants with unclear workplace affiliation (*n* = 53). Data were collected by a data collection company (Enkätfabriken) via a web survey, with individual survey links distributed by the unions to the registered email addresses of eligible midwives. The first round of data collection took place between February and April 2020, with a follow-up survey conducted in 2023 between April and May and between September and October. Three reminders were sent to encourage participation. The data collection company generated and managed the database used for analysis.

Participants were matched across the two survey waves using a unique project-specific identification code generated by each respondent at baseline and reproduced at follow-up. This code allowed longitudinal linkage without accessing personal identifying information.

Attrition analysis was conducted to assess potential bias between respondents who participated in both survey waves and those who only participated in 2020. Differences in age, years of experience, care setting (primary vs hospital care), and baseline intention-to-leave scores were examined using chi-square tests and independent samples *t*-tests. No significant differences were identified across these variables, indicating that attrition was unlikely to have introduced systematic bias in the longitudinal sample.

### 2.2 Measures

Single-item measures were used for intention outcomes. Prior research shows that single-item indicators perform acceptably when constructs are narrow, concrete and clearly defined [38], [39], [40]. Turnover intention has frequently been measured using single items in organisational and turnover research, with evidence demonstrating adequate validity and predictive utility [41]. These items were also used in previous GoodWEM studies and demonstrated face validity in pilot testing [37].

Intention to leave position was measured with the item: *Have you considered leaving your current position as a midwife?*. The question ranged from 0 to 100, with higher scores indicating a greater intention to leave.

Intention to leave the profession was assessed with the item: *Have you considered stopping working as a midwife?*

Actual turnover was defined as no longer working at the same workplace as in 2020 at the time of the 2023 follow-up. Respondents who remained in the same workplace were coded as 0.

Perceived satisfaction with salary level: Participants rated their satisfaction with their current salary level by answering a question regarding “How satisfied are you with your salary?”. The question ranged from 0 to 100, with higher scores indicating greater satisfaction.

Demographic variables: Years of experience and type of care (primary care vs. hospital care) were analysed for their potential influence on the outcomes.

### 2.3 Statistical analyses

Changes in intention to leave and salary satisfaction between 2020 and 2023 were analysed using paired *t*-tests. Descriptive statistics were reported as means and standard deviations (M ± SD) for continuous variables and frequencies with percentages (n, %) for categorical variables. Linear regression analyses were conducted to evaluate the associations between changes in intention to leave, salary satisfaction, and demographic variables. Logistic regression models were used to examine predictors of [1] increased intention to leave the profession, and actual turnover. Regression models were adjusted for age, years of experience, care setting (primary vs hospital), and baseline intention scores. All statistical analyses were conducted in R (version 4.5.2) via RStudio (version 25.09.2) by the statistician in the research team.

## 3 Results

The participants' ages ranged from 22 to 65 years (mean age = 28.5 years, SD = 9.9), and they had an average of 13.9 years of professional experience (SD = 10.3), 38.5% (n = 179) worked in primary care, while 61.5% (n = 286) were employed in hospital care, see Table 1. In 2023, 39% of midwives reported considering leaving the profession, and it was 40% that had changed positions during the study period from 2020 to 2023, Table 1.

**Table 1:** Sample characteristics and change in intention to leave and in the perceived satisfaction with salary level from 2020 to 2023.

	2020	2023	∆ 2020–2023	
	M (SD)/n (%)	M (SD)/n (%)	M (SD)/n (%)	p*
Age, M (SD)	28.9 (9.7)			
Experience, M (SD)	14.5 (10.3)			
Primary care, n (%)	192 (40.2)			
Hospital care, n (%)	286 (59.8)			
Intention to leave the position, M (SD)	40.2 (30.0)	36.6 (28.8)	-3.6 (31.7)	.013
Intention to leave profession, m (SD)		187 (39.2)		
Actual turnover, n (%)		192 (40.2)		
Salary level, M (SD)	35.0 (27.7)	42.6 (28.9)	7.6 (29.6)	< .001

* Derived from dependent *t*-test

### 3.1 Predictors of changes in intention to leave their position

In 2020, midwives had a baseline intention to leave their position of 40 points. Changes in Intention to leave the position between 2020 and 2023 were significantly predicted by baseline Intention to leave the position in 2020, baseline salary satisfaction in 2020, and changes in salary satisfaction, Table 2. Professional experience was also a significant predictor, with greater experience associated with smaller changes in Intention to leave the position. Workplace setting did not significantly influence changes in intention to leave. These variables explained 54.9% of the variance in changes in Intention to leave the position.

**Table 2:** Linear regression analysis of the associations of change in intention to leave and change in the perceived satisfaction with salary level from 2020 to 2023.

	B	S.E.	β	p	∆ R^2^
**Change in Intention to leave the position**					
Experience	-0.347	0.105	-0.113	< .001	.008
Salary level 2020	-0.145	0.045	-0.128	< .001	.002
Change in salary level	-0.218	0.039	-0.206	< .001	.064
Intention to leave the position 2020	-0.731	0.036	-0.698	< .001	.329
Intention to leave profession	25.607	2.131	0.399	< .001	.144
Actual turnover 2020–2023	2.929	2.269	0.044	.197	.002
Primary care	-0.386	2.097	-0.006	.854	.000
R^2^	.549				
**Change in Salary level**					
Experience	0.104	0.121	0.036	.392	.013
Salary level 2020	-0.578	0.045	-0.540	< .001	.249
Intention to leave the positionIntention to leave the position 2020	-0.215	0.056	-0.218	< .001	.001
Change in intention to leave the positionIntention to leave the position	-0.285	0.051	-0.302	< .001	.072
Intention to leave profession	-2.296	2.789	-0.038	.670	.001
Actual turnover 2020–2023	0.322	2.601	0.005	.902	.000
Primary care	-7.162	2.377	-0.119	.003	.013
R^2^	.336				

Note: B = unstandardised regression coefficient; β = standardised regression coefficient; S.E. = standard error; R² = coefficient of determination; ∆ R^2^ = change in coefficient of determination.

### 3.2 Predictors of changes in perceived salary satisfaction

The salary satisfaction increased significantly from 2020 to 2023. Baseline salary satisfaction in 2020 was the strongest predictor of changes in perceived salary satisfaction, Table 2. Changes in Intention to leave the position and baseline Intention to leave the position in 2020 were also significant predictors. Working in hospital care was positively associated with changes in perceived salary satisfaction. Neither professional experience nor actual turnover during the study period significantly predicted changes in salary satisfaction. The model explained 33.6% of the variance in changes in perceived salary satisfaction.

### 3.3 Predictors of intention to leave profession

Logistic regression analysis showed that baseline Intention to leave the position in 2020 and changes in Intention to leave the position from 2020 to 2023 were significant predictors of intention to leave the profession in 2023, Table 3. Neither professional experience, salary satisfaction, changes in salary satisfaction, nor workplace setting were significant predictors. The model demonstrated strong predictive capacity, with Cox & Snell R² = 0.314 and Nagelkerke R² = 0.426.

**Table 3:** Logistic regression of the associations with intention to leave profession in 2023 and actual turnover between 2020 and 2023.

	B	S.E.	OR (95% CI)	p	∆ R^2^
**Intention to leave profession in 2023**					
Experience	0.024	0.013	1.024 (0.99, 1.05)	.064	.003^a^,.005^b^
Salary level	-0.009	0.005	0.991 (0.98, 1.00)	.086	.032,.042
Change in salary level	-0.003	0.005	0.996 (0.99, 1.01)	.426	.035,.048
Intention to leave the positionIntention to leave the position 2020	0.054	0.006	1.055 (1.04, 1.07)	< .001	.044,.060
Change in intention to leave the positionIntention to leave the position	0.052	0.006	1.053 (1.04, 1.07)	< .001	.198,.267
Actual turnover 2020–2023	0.357	0.267	1.429 (0.85, 2.41)	.182	.002,.003
Primary care	-0.151	0.250	0.860 (0.53, 1.40)	.545	.000,.001
-2 Log Likelihood	457.556				
Cox & Snell R²	.314				
Nagelkerke R²	.426				
**Actual turnover between 2020 and 2023**					
Experience	-0.092	0.013	0.912 (0.89, 0.94)	< .001	.097^a^,.134^b^
Salary level	0.018	0.005	1.018 (1.01, 1.03)	< .001	.028,.030
Change in salary level	-0.001	0.004	0.977 (0.99, 1.01)	.977	.003,.004
Intention to leave the positionIntention to leave the position 2020	0.005	0.005	1.007 (0.99, 1.01)	.334	.000,.000
Change in intention to leave the positionIntention to leave the position	0.007	0.005	1.005 (0.99, 1.02)	.160	.009,.012
Intention to leave profession	0.289	0.266	1.335 (0.79, 2.25)	.276	.002,.002
Primary care	1.039	0.232	2.827 (1.80, 4.45)	< .001	.037,.052
-2 Log Likelihood	512.206				
Cox & Snell R²	.176				
Nagelkerke R²	.245				

Note: B = logistic regression coefficient (log-odds); S.E. = standard error; OR = odds ratio; CI = confidence interval; p = p-value; ΔR² = change in explained variance (pseudo-R²) between models; –2 Log Likelihood = model deviance, with lower values indicating better model fit; Cox & Snell R² and Nagelkerke R² = pseudo-R² statistics indicating the proportion of variance explained by the model.^a^Cox & Snell R², ^b^Nagelkerke R²

### 3.4 Predictors of actual turnover (2020–2023)

Logistic regression analysis revealed that workplace setting was a significant predictor of actual turnover, with midwives working in primary care being more likely to change positions than those in hospital care, Table 3. Professional experience was negatively associated with turnover. Higher baseline salary satisfaction in 2020 was positively associated with turnover, suggesting that those with greater salary satisfaction in 2020 were more likely to change roles during the study period. The model explained 17.6% of the variance in turnover based on Cox & Snell R² and 24.5% based on Nagelkerke R².

## 4 Discussion

The aim of this study was to analyse changes in midwives' intentions to leave their position, intentions to leave the profession, actual turnover rates, and salary satisfaction over three years in Sweden. A secondary aim was to investigate differences between primary and hospital care settings and predictors of turnover.

Four key findings emerged, first 39% of the midwives had an intention to stop working as a midwife and leave the midwifery profession. Second, baseline intention to leave in 2020, salary satisfaction, and changes in salary satisfaction were significant predictors of changes in intention to leave their position. Third, baseline intention to leave their position and changes in intention to leave significantly predicted intention to leave midwifery. Fourth, midwives in primary care were more likely to change positions than those in hospital care.

An important conceptual contribution of this study is the clear distinction between intention to leave the position and intention to leave the profession. These represent different psychological processes: leaving a position typically reflects dissatisfaction tied to local workplace conditions, whereas leaving the profession indicates deeper concerns about role sustainability and long-term career viability [22], [24], [41]. Our findings on the differing predictors for these outcomes reinforce this theoretical distinction. The alarming findings of this study, that 39% of midwives expressed an intention to leave the profession, highlighting a significant workforce challenge. A recent article from the GoodWEM project identified key determinants of midwives’ retention including professional recognition, development opportunities and health. Conversely, burnout was linked to a higher likelihood of leaving the profession [37].

This finding aligns with Psychological Contract Theory, which suggests that when expectations related to support, recognition or career development are perceived as unmet, employees may experience a breach of the psychological contract, increasing withdrawal behaviours [27], [28]. Social Exchange Theory further supports this interpretation, proposing that insufficient perceived reciprocity from the organisation may diminish commitment and strengthen intentions to leave [29], [30].

This study confirmed that salary satisfaction influences midwives' intentions to stay or leave, aligning with existing literature on salary as a determinant of retention [10], [14], [26], [42]. Changes in salary satisfaction predicted changes in intention to leave is supported by research emphasising the psychological impact of perceived fairness and financial adequacy in fostering workforce stability [14], [21].

These findings are consistent with Social Exchange Theory, wherein fair compensation is interpreted as organisational reciprocity and fosters stronger commitment [30]. Improvements in salary satisfaction between 2020 and 2023 may therefore have contributed to the observed reduction in intention to leave the position.

However, the present results also showed that salary satisfaction did not significantly predict intention to leave midwifery, highlighting the complex interplay of factors influencing professional attrition. Previous studies have shown that midwives' decisions to leave the profession are often driven by broader psychosocial factors, such as perceived organisational support and professional fulfilment, rather than salary alone [5], [7], [8], [9], [36]. This underscores the importance of also addressing non-financial aspects of job satisfaction, including mentorship and career development opportunities, particularly for early-career midwives [11], [13].

The lack of significant influence of workplace setting on intention to leave contrasts with research suggesting that factors such as working hours, workload, and work schedules play a key role in midwives’ job satisfaction [21], [43]. The current findings indicate that workplace setting alone may not fully account for the complex factors influencing midwives’ intentions to leave, particularly within the Swedish healthcare context.

However, the substantially higher actual turnover among primary care midwives suggests the presence of structural or organisational factors, such as a more fragmented employment landscape, greater job mobility, or differing expectations of role autonomy, that may facilitate job changes independent of stated intentions. This discrepancy highlights the importance of analysing both intention and behaviour when assessing workforce stability.

The finding that higher salary satisfaction was associated with a greater likelihood of turnover diverges from previous research [25], [26]. A plausible explanation within the Swedish labour market is that salary increases are often achieved through changing employers rather than through internal progression. Midwives who recently changed workplace may therefore report higher salary satisfaction because the move resulted in a higher wage, making elevated satisfaction a reflection of recent job mobility rather than an indicator of retention. In this context, higher salary satisfaction and turnover may coincide, as salary growth frequently occurs through external job changes in the healthcare sector. A strength of this study is the nationwide sample of midwives and the longitudinal design, which provides knowledge regarding changes in workforce dynamics over time, a perspective lacking in many cross-sectional studies. Additionally, the inclusion of both primary and hospital care settings allows for a nuanced comparison of work environments.

However, the study also has limitations. A key limitation of this study is the reduced sample size, as only 478 midwives participated in both the 2020 and 2023 surveys, representing a subset of the original national cohort of 2060 midwives in the GoodWEM study. While attrition analysis indicated no significant differences between included and excluded participants, the loss of eligible respondents, particularly those with unclear workplace settings, may limit the generalisability of the findings.

Another limitation is the exclusive reliance on survey data, without any qualitative follow-up to contextualise why midwives consider leaving their position or profession. Qualitative methods could illuminate underlying motivations, contextual pressures and career trajectories.

The use of single-item measures for intention outcomes may also limit depth of construct measurement. However, single-item intention items are widely used in turnover research when constructs are narrow, concrete and easily interpreted [37], [38], [39], [40], [41].

Additionally, as a longitudinal study relying on survey data, potential response bias and unmeasured confounders should be considered when interpreting the results. The reliance on self-reported measures introduces the potential for response bias, as participants may have under- or overestimated their intentions to leave or satisfaction levels.

Another limitation of the study was its reliance on distribution through unions, thereby excluding midwives who are not union members. Although the vast majority of Swedish midwives working in healthcare are affiliated with the two included unions, reducing the potential for selection bias arising from this approach.

## 5 Conclusions

The high proportion of midwives considering leaving underscores the urgency of addressing workplace conditions and support systems. Ensuring sustainable workloads, enhancing professional recognition, and promoting midwives' health and wellbeing are critical strategies to reduce turnover and ensure the long-term stability of the profession. Future research should further examine organisational and leadership prerequisites to develop targeted interventions that effectively support midwives' retention.

This study identifies key factors influencing midwives’ intentions to leave their roles and the profession within Swedish healthcare. Given midwives’ vital role in maternal and neonatal health, the findings underscore the need for targeted efforts to improve working conditions, ensure fair pay relative to responsibility and education, and support workforce retention. Strengthening midwifery services in this way is essential for sustaining their contribution to public health and improving outcomes for mothers and newborns.

## CRediT authorship contribution statement

**Marcus Praetorius Björk:** Writing – original draft, Visualization, Resources, Methodology, Formal analysis, Conceptualization. **Lena Henriksen:** Writing – original draft, Methodology. **Maimburg Rikke:** Writing – original draft, Methodology. **Malin Hansson:** Writing – original draft, Project administration, Methodology, Investigation, Funding acquisition, Conceptualization.

## Consent for publication

Not applicable.

## Ethics approval and consent to participate

The Swedish Ethical Review Authority, department of Umeå, approved the study in 2019 (Dnr 2019–03776) and it was performed in accordance with the Declaration of Helsinki from 1964 and its later amendments regarding research involving human subjects. All participants were assured of confidentiality and anonymity. The participants gave informed consent before taking part in the study.

## Declaration of Generative AI and AI-assisted technologies in the writing process

During the preparation of this work the author used ChatGPT in order to improve the language and readability of the manuscript. After using this tool/service, the authors reviewed and edited the content as needed and takes full responsibility for the content of the publication.

## Funding

This work was supported by research grants from 10.13039/501100002706AFA Insurance (no. 210163). The study was also financed by grants from the Swedish state under the agreement between the Swedish government and the county councils, the ALF-agreement (ALFGBG-984421).

## Declaration of Competing Interest

The authors have no financial or other relationships to declare which might lead to competing interest.
